# Type B Aortic Dissection Management: A Narrative Review of Guidelines and Systematic Reviews

**DOI:** 10.31661/gmj.v12i.2967

**Published:** 2023-12-18

**Authors:** Samir Ghimire, Arman Arghami, Aresha Masood Shah, Marium Billoo, Rehan Billoo, Mohammad Zarenezhad, Asra Iqbal, Somayeh Ahmadnezhad, Fatemeh Maleki, Behrang Rezvani Kakhki, Sayyed Majid Sadrzadeh, Somayeh Mehrpour, Roohie Farzaneh, Uzair Yaqoob

**Affiliations:** ^1^ Carribean Medical University, Willemstad, Curacao; ^2^ Cardiovascular Surgery Department, Mayo Clinic, Rochester, Minnesota, USA; ^3^ Jinnah Postgraduate Medical Centre, Karachi, Pakistan; ^4^ Legal Medicine Research Center, Legal Medicine Organization, Tehran, Iran; ^5^ Ramsar Campus, Mazandaran University of Medical Sciences, Ramsar, Iran; ^6^ Department of Emergency Medicine, Faculty of Medicine, Birjand University of Medical Sciences, Birjand, Iran; ^7^ Department of Emergency Medicine, Faculty of Medicine, Mashhad University of Medical Sciences, Mashhad, Iran; ^8^ Department of Anesthesiology & Critical Care, Qom University of Medical Sciences, Iran; ^9^ Department of Neurosurgery, Civil Hospital, SMBBIT, Karachi, Pakistan

**Keywords:** Aortic Dissection, Endovascular Repair, Review, Systematic Review, Meta-analysis

## Abstract

Background: Surgical or medical treatment for type B or descending aortic dissections with difficult presentation or stable hemodynamics is debatable. This study aimed to review the type B aortic dissection therapy to assess safety and effectiveness. Materials and Methods: Online databases of PubMed, Science Direct, Web of Science, Cochrane, and Scopus were searched for relevant systematic reviews, guidelines, and meta-analysis studies on the management of type B aortic dissection, up to July 2023. The conclusions were qualitatively synthesized. Results: Best medical therapy (BMT], thoracic aortic endovascular repair (TEVAR), and open surgeries (OS) were management approaches. Hemodynamics classify type B aortic dissection as complex or simple. Both examples reveal decreased in-hospital all-cause mortality with TAVR than OS. Guidelines recommend TEVAR for difficult situations and OS if it fails. Complication analyses favour TEVAR, however left subclavian artery coverage without revascularization increases stroke risk. Studies show Type B aortic dissection is simpler than TEVAR and BMT. Acute or subacute presentation did not affect reintervention rates between treatments. TEVAR had a greater early stroke risk than BMT but a decreased long-term aortic-related and all-cause mortality. The best data showed no differences in in-hospital mortality or early re-intervention between regimens. BMT reduced early stroke but increased late all-cause death. Conclusion: In conclusion, addressing Type B aortic dissection is complicated, depending on presentation and hemodynamics. TEVAR is best for difficult patients, however BMT and OS also work. TEVAR may reduce in-hospital mortality but increase early stroke risk.

## Introduction

Aortic dissection is an emergency in which separation of the intima of the aorta causes the dissection of blood into the vessel wall and is almost always characterized by a luminal tear [1]. Aortic dissection is an uncommon disease and its reported rate is 5 to 27 cases per 100,000 people [2]. It is a life-threatening condition and delayed diagnosis and treatment is associated with high mortality [1]. It is estimated that the mortality rate increases by one percent for every hour of delay in treatment. But with early diagnosis and timely treatment, the survival rate of this condition increases significantly [3]. More than 25% of untreated people die within the first 24 hours, 50% within the first week and more than 75% within the first month. Rapid diagnosis and management of this disease is the key to reducing morbidity and mortality [4]. It is estimated that 21% of patients with aortic dissection die before treatment in the hospital [2]. This disease is divided into two types, type A and type B in the Stanford classification [5]. In type A, the definitive treatment is surgery. Type B includes the involvement of the descending aorta, whose primary strategies are medical or interventional treatments [5]. A systematic review and meta-analysis compared thoracic endovascular aortic repair (TEVAR] and open chest surgical repair (OCSR) for type B aortic dissection (TBAD) in 18 studies. TEVAR showed superior short-term survival benefits with reduced in-hospital mortality compared to OCSR. TEVAR also demonstrated enhanced safety, presenting lower risks of complications [6]. TEVAR is advancing rapidly and finds applications in various aortic conditions. However, as more data emerges, there are indications of increased reoperation rates associated with TEVAR [7]. The latest guideline by the Society of Thoracic Surgeons/American Association for Thoracic Surgery in 2022, recommends that in cases of TBAD with progression of disease despite optimal medical therapy (OMT) and in patients with connective tissue disorders, open surgical repair may be a reasonable alternative to TEVAR, as it offers greater durability in treatment [8].

The goal of this study is to conduct a narrative review on the management of type B aortic dissection or dissection of the descending aorta. The study aims to address the existing controversies and differences in opinion regarding the best treatment strategy for type B or descending aortic dissections, particularly in cases of complicated presentation or stable hemodynamics. By summarizing and analyzing the findings from multiple pieces of evidence, this research intends to provide a comprehensive overview of the current evidence on type B aortic dissection therapy. While some studies have reported favorable outcomes with specific treatment modalities, others have questioned their long-term effectiveness and safety. This study would examine and synthesize the findings from various studies to identify areas of consensus.

## Materials and Methods

The current study is a narrative review of aortic dissection surgical management is reviewed. To ensure specific and well-defined research objectives, the PICO (Population, Intervention, Comparison, Outcome) questions were used to formulate the study.

P: Patients diagnosed with type B aortic dissection (uncomplicated or complicated]. I: Different types of surgical (thoracic aortic endovascular repair (TEVAR), and open surgeries (OS) and non-surgical management (Best medical therapy (BMT) approaches. C: Comparisons between the various management methods. O: Short-term and long-term clinical outcomes. So, the study question is "Among patients diagnosed with type B aortic dissection, what are the comparative short-term and long-term clinical outcomes, including survival rate and complications or reintervention, for those treated with different management approaches, such as thoracic aortic endovascular repair (TEVAR), open surgeries (OS), and best medical therapy (BMT)?".

Based on the presented questions, keywords were selected for a comprehensive review of the literature in databases of PubMed, Scopus, Elsevier‎, Web of Science, and Cochrane databases and the google scholar search engine. Logical combinations of the keywords of "aortic dissection ", "type B", "endovascular repair", "type B aortic dissection", "descending aortic dissection", "open chest surgical repair", "Stent-Graft Placement", "thoracic aortic endovascular repair", "optimal medical therapy", "best medical therapy", "open chest surgery", "open surgery" by the operator of OR along with keywords of the "systematic", ("systematic review", "review", "meta-analysis", "guideline" using the AND operator was searched. There was no time restriction and all publications before July 2023 were searched. studies and searches were restricted to English-language publications. Two independent authors performed the search strategy separately and primary records were screened for removing duplicated papers.

The inclusion criteria for this study were systematic review, guideline, and meta-analysis studies. Meta-analysis was not a prerequisite during the study selection process. Only acute cases were included and chronic TBAD was not considered. Systematic reviews about TBAD in certain populations like Marfan disease were not considered. Studies had to be about management methods of acute TBAD management, not risk factors or prognostic risk factors. Only studies on type B aortic dissections were included. ‎ Studies on aneurysms without dissection were not included. ‎ Aortic dissections out of the chest and thorax were not considered. So, systematic review studies including abdominal aortic dissection were not included. Two independent authors evaluated studies for eligibility for inclusion in the study and any disagreement between them was resolved by the third author. In the first step, separate articles from different databases were collected, and then based on the bibliography and abstracts, duplicate papers were removed. In the next step, three steps, by reading, the title, abstract and full text of the articles, irrelevant articles were removed according, to both authors. Data extraction from the articles that entered the final review was also done through a prespecified checklist including the number of studies included in the systematic review, the last date searched, study designs of the included studies, comparisons, interventions, and outcomes. Qualitative evidence synthesis was performed by comparing the similar outcomes of systematic reviews with each other. Quantitative synthesis was not performed as there was a high overlap between the studies.

## Results

**Table:** Table1. Characteristics Included Systematic Review and Mta-analysis Studies

	study design	n of included studies	study design of included studies	Comparison/groups	follow up length
Uncomplicated type B aortic dissection		
Wang et al., 2022 [9]	SR-MA	11	RCT or retrospective	TEVAR vs. BMT	1 to 60 months
Hossack et al., 2020 [10]	SR-MA	8	RCT		
Yang et al., 2022 [11]	SR-MA	3	Retrospective	acute vs. subacute TEVAR	up to 3 years
Merola et al., 2013 [12]	SR	7	Mixed	TEVAR vs. BMT	2 years
Uncomplicated/Complicated type B aortic dissection	
Harky et al., 2019 [13]	SR-MA	9	mixed retrospective and prospective	TEVAR vs. OS	1 year
Liu et al., 2019 [14]	SR-MA	18	mixed retrospective and prospective	TEVAR vs. OS	1-5 year
Li et al., 2018 [15]	SR-MA	15	mixed retrospective and prospective	TEVAR vs. OS	NA
Hao et al., 2012 [16]	SR-MA	5	mixed retrospective and prospective	TEVAR vs. OS	NA
Liu D et al., 2020 [17]	SR-MA	18	mixed retrospective and prospective	TEVAR vs. OS vs. BMT	1-5 year
Zhu et al., 2016 [18]	SR-MA	9	Prospective	TEVAR vs. OS	5 years
Wilson-Smith et al., 2021 [19]	SR-MA	46	Prospective	TEVAR	10 years
Luebke and Brunkwall, 2010 [20]	SR-MA	76	case series and retrospective	TEVAR	1 to 10 years
left subclavian artery LSA ostial coverage
Karaolanis et al., 2022 [21]	SR-MA	43	Retrospective	TEVAR	Varying
Guidelines
STS/AATS, 2022 [22]	Panel of experts and SR	NR	Mixed	stepwise evaluation & treatment for TBAD, emphasizing BMT & appropriate surgical revascularization interventions; LSA revascularization to prevent spinal cord ischemia.	NA
ESVS, 2017 [23]	Panel of experts and SR	NR	Mixed	Comparison of all methods of BMT, TEVAR, and OS	NA
German clinical practice guidelines, 2023 [24]	Panel of experts and SR	NR	Mixed	Comparison of all methods of BMT, TEVAR, and OS	NA
Society for Vascular Surgery, 2021[25]	Panel of experts and SR	NR	Mixed	Comparison of all methods of BMT, TEVAR, and OS	NA


**BMT**; Best medical therapy, **TEVAR**; thoracic aortic endovascular repair, **OS**; open surgeries, **STS/AATS**; The Society of Thoracic Surgeons/American Association for Thoracic Surgery, **ESVS**; European Society for Vascular Surgery, **NR**; not reported, **NA**; not applicable

**Figure-1:**
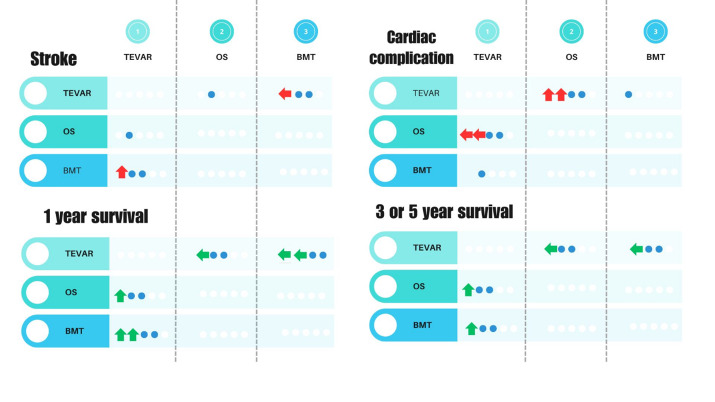
Figure 1. Qualitative summary of pairwise comparison of pooled complications and survival in evaluated studies. Circles are representative of studies, where white circles stand for studies with no statement about the comparison, blue ones as no significant difference in comparison, and arrows showing higher odds in the directed group.

Table-1, shows the included studies [6][8][9][10][11][12][13][14][15][16][17][18][19][20][21][22][23]. Best medical therapy (BMT), thoracic aortic endovascular repair (TEVAR), and open surgeries (OS) are used for the management of this condition in different trials.

Type B aortic dissection is categorized as complicated or uncomplicated based on hemodynamics. In both complicated and uncomplicated cases, TAVER had 0.19 to 0.54 times lower odds of in-hospital all-cause mortality compared with the OS, based on the review of 5 studies (Harky et al., 2019, Liu et al., 2019, Li et al., 2018, Hao, Liu D, et al., 2020). TAVR is a less invasive procedure compared to traditional open surgery, which requires a larger incision to access the aorta; so, the results are showing better short-term outcomes in TEVAR than the OS. But long-term efficacy should also be investigated. As shown in Figure 2, one study stated that TEVAR-treated patients had better one-year survival than OS patients; while other studies did not find any difference. Long-term survival (3 or 5 years) was higher in TEVAR than in BMT groups only in one study. One study pooled survival statistic of only TEVAR patients in 46 papers and found that the survival rate for all 1, 2, ,4, and 8 years was higher than 60% for these patients (Wilson-Smith et al., 2021). Another meta-analysis had similar findings of lower short-term mortality in TEVAR than open surgical patients while no differences in complications and late mortality rates (Luebke and Brunkwall, 2010) as well as what was found in Zhu et al. study (Zhu et al., 2016). Considering all these findings together, in complicated cases, TEVAR is the first preferred line of treatment. Society for Vascular Surgery also confirmed this [21]. OS might be needed in case of failure of TEVAR based on the ESVS guidelines [23].

Comparing TEVAR versus OS for complications, OS-treated patients had higher neurologic, cardiac, and renal complications based on the Harky et al. study while Liu J et al., Li et al., Hao et al., and Liu D et al. showed that there were no significant differences in the rate of the most complications in their comparisons between TEVAR, OS, and BMT, except for some statistical differences that were seen in Liu J study that renal and pulmonary complications were higher in OS than the TEVAR. In the case of stroke in TEVAR patients, the risk is particularly high for patients who have their left subclavian artery (LSA) covered during the procedure without revascularization (restoring blood flow to the LSA). Revascularization of the LSA is recommended to reduce the risk of stroke in these cases [19]. Based on the STS/AATS, this stroke risk reduction involves ensuring that blood flow to the LSA is maintained or restored using appropriate techniques, which can help reduce the chances of spinal cord ischemia occurring as a complication of the TEVAR procedure [20]. ESVS suggests LSA revascularization in a ruptured TBAD in an anatomy of bypass of the left mammary artery to the coronary artery or the augmentation of cerebral blood supply from the dominant left vertebral artery [21]. A summary of the qualitative findings of the review studies is presented in Figure-1.

Figure 1. Qualitative summary of pairwise comparison of pooled complications and survival in evaluated studies. Circles are representative of studies, where white circles stand for studies with no statement about the comparison, blue ones as no significant difference in comparison, and arrows showing higher odds in the directed group.

Uncomplicated Type B Aortic Dissection

Uncomplicated Type B dissections are referred to conditions in which patients have stable hemodynamic situations. We identified 4 systematic review and meta-analysis studies on the uncomplicated thoracic aortic type B dissection with uncomplicated patients. Wang et al. compared the BMT versus TEVAR in 11 trials [9]; while there were duplicated individual patients in 3 studies that were belonging to a single cohort at different follow-up intervals. Merola et al. did the same 10 years ago with 6 studies (123 TEVAR versus 566 BMT patients) [12]. Yang et al. study in the same year was performed with stratification of patients based on the manifestation severity as acute and sub-acute based on the European Society for vascular surgery (ESVS) guideline, with 718 acute and 457 sub-acute participants [11]. Yang et al. studies included reports that had different definitions of acute or sub-acute but most were referring to acute as occurring within 1 month of symptoms. Li et al. study was a systematic review and meta-analysis of both complicated and uncomplicated cases, individually [14]. While the German clinical practice guidelines suggest that patients with acute uncomplicated should be treated with TEVAR in the subacute phase [22].

The qualitative review showed that reintervention rates were not statistically different between TEVAR and BMT for uncomplicated type B ones in both Merola et al. and Wang et al. studies. Presenting acute or sub-acutely does not seem to affect the reintervention rate based on Yang et al. (low-quality evidence). In case of complications, End-organ damage was only significantly higher presenting in TEVAR-managed patients than in BMT-managed patients in Merola et al. study in two years of follow-ups (low-quality evidence). In Wang et al. study with moderate quality of evidence, early stroke was higher in TEVAR-managed patients than BMT-managed patients; but in the long term, rupture, reintervention, aortic-related death, and all-cause mortality was higher in patients treated with BMT than TEVAR. Also, TEVAR increases the chance of thoracic false lumen thrombosis.

In the highest level of evidence for uncomplicated TBAD, a meta-analysis of RCT studies showed that there were no significant differences between TEVAR and BMT in terms of in-hospital mortality, early re-intervention by TEVAR, or surgery. However, BMT demonstrated a significantly lower risk of early stroke. Conversely, BMT was associated with a higher risk of late all-cause mortality [10].

Based on Li et al., late aneurismal dilatation is more prevalent in patients undergoing BMT than in patients treated with TEVAR (non-explained high heterogenicity and low quality of evidence). Based on Yang et al., these findings in TEVAR patients are not affected by the acute or subacute presentation of the disease.

## Discussion

Our study found that in-hospital all-cause mortality of patients with type B aortic dissection with TEVAR is lower than OS. This finding is robust and supported by frequent observations with a low risk of bias. In patients presenting with suspected symptoms of aortic dissection, after validation of the diagnosis based on the available methods of the computed tomography (CT) scans, magnetic resonance imaging (MRI), angiography, transthoracic echocardiography (TTE), and transesophageal echocardiography (TEE), guidelines suggest different practical management methods based on the patient’s medical condition. In case of complicated aortic dissection presenting with hypoperfusion and unstable hemodynamics, surgery would be inevitable that open surgical approach is proposed for the larger than 40 mm aortic diameter [24]; but after Dake et al. study in 1994 [25], the TEAVR use has been increasingly preferred. There were only controversies in the long-term safety and efficacy of TEAVR compared to open surgery our review found that there is no evidence of higher complications in TEAVR than the open surgery and long-term survival did not show any significant differences. So, there is robust evidence that complicated type B aortic dissection should be managed by the TEAVR.

In the case of uncomplicated type B aortic dissection, the best medical therapy is suggested on the potential risks of late complications of the aortic dissection. Best medical therapy is defined as keeping systolic blood pressure under 120 mmHg and heart rate lower than 70 beats per minute, first by intravenous blood pressure-lowering medications such as beta-blockers or alpha-blockers and then oral regimens [26]. Our review found that TEAVR would be better for uncomplicated type B dissection due to the risk of long-term rupture, aortic-related death, and all-cause mortality is higher in BMT-treated patients than TEAVR, based on the summary of all reviewed studies. As well as our study, a narrative review study by Jubouri et al. in 2022 demonstrated that the first choice for even uncomplicated TBAD is TEVAR for achieving the best survival outcomes [27]. But, some individual RCT studies, like the INvestigation of STEnt Grafts in Aortic Dissection (INSTEAD) trial, show that compared to BMT, TEVAR did not lead to an improvement in survival rates and adverse events [28]. Then in another trial in 2013, the INSTEAD-XL trial, TEVAR along with the BMT increased the survival rates [29]. While in the primary trial of the Acute Dissection Stent Grafting or Best Medical Treatment (ADSORB), the BMT+ TEAVR group had better survival compared to the BMT-only group [30]. So, the composite of approaches being used for the treatment would also affect the outcome. Hybrid interventions involve a combination of BMT, endovascular stent graft placement, and open surgical procedures. Typically, the primary entry tear in TBAD is situated near the orifice of the LS). For successful TEVAR with the goal of closing the primary entry in Type B dissection, it is crucial to ensure a secure proximal landing zone in the aortic arch. Achieving this often necessitates a hybrid surgical approach that involves incorporating open surgical techniques, like debranching, to revascularize the cervical branches [31].

Our study’s main limitation is the high overlap among the studies. All studies included in Harky et al. meta-analysis were also included in the liu J et al. study but different populations from Conrad et al. study [32] were used in analyses. Also, there are few randomized studies and all evidence synthesized in the included systematic review and meta-analysis studies is about non-randomized patients.

## Conclusion

In conclusion, the management of Type B aortic dissection presents a challenging clinical scenario, and the approach to treatment varies depending on the presentation and hemodynamic status of the patient. The available evidence from systematic reviews, guidelines, and meta-analyses supports several management approaches.

1-For complicated Type B aortic dissections, TEVAR is the preferred treatment, as it has shown lower in-hospital all-cause mortality compared to OS. However, it is important to consider the risk of stroke when the left subclavian artery is covered without revascularization during TEVAR. In cases where TEVAR is not feasible or has failed, OS can be considered as an alternative treatment option.

2-For uncomplicated Type B aortic dissections, studies have compared TEVAR and BMT, showing similar reintervention rates irrespective of acute or subacute presentation. While TEVAR carries a higher early stroke risk, it also demonstrates a lower long-term risk of aortic-related death and all-cause mortality compared to BMT. On the other hand, BMT has a lower risk of early stroke but a higher risk of late all-cause mortality.

The highest level of evidence indicates no significant differences in in-hospital mortality or early re-intervention between TEVAR and BMT. However, the choice of treatment should be carefully considered based on individual patient characteristics and the specific circumstances of the Type B aortic dissection.

In conclusion, managing Type B aortic dissection requires a tailored approach, taking into account the complexity of the condition and the patient's hemodynamic status. TEVAR is generally favored for complicated cases due to its favorable outcomes, while BMT and OS remain relevant treatment options in certain scenarios. The evidence suggests that TEVAR may offer lower in-hospital mortality but carries a higher early stroke risk. Ultimately, a multidisciplinary approach and careful consideration of available evidence are essential in determining the most appropriate management strategy for each patient.

## Conflict of Interest

None.
